# Fast-Track Surgery Improves Postoperative Clinical Recovery and Immunity After Elective Surgery for Colorectal Carcinoma: Randomized Controlled Clinical Trial

**DOI:** 10.1007/s00268-012-1606-0

**Published:** 2012-04-24

**Authors:** Dongjie Yang, Weiling He, Sheng Zhang, Huayun Chen, Changhua Zhang, Yulong He

**Affiliations:** Department of Gastrointestinal and Pancreatic Surgery, The First Affiliated Hospital of Sun Yat-sen University, 58 Zhongshan Rd II, Guangzhou, 510080 China

## Abstract

**Background:**

Few clinical studies or randomized clinical trial results have reported the impact of fast-track surgery on human immunity. This study aimed to investigate the clinical and immune impact of fast-track surgery in colorectal cancer patients undergoing elective open surgery.

**Methods:**

A controlled randomized clinical trial was conducted from November 2008 to January 2009 with a 1-month postdischarge follow-up. A total of 70 patients with colorectal carcinoma requiring colorectal resection were randomized into two groups: a fast-track group (35 cases) and a conventional care group (35 cases). All included patients underwent elective open colorectal resection with combined tracheal intubation and general anesthesia. Clinical parameters and markers of immune function were evaluated in both groups postoperatively.

**Results:**

In all, 62 patients completed the study: 32 in the fast-track group and 30 in the conventional care group. Our findings revealed a significantly shorter postoperative hospital stay and faster return of gastrointestinal function in patients undergoing fast-track rehabilitation. In addition, we found a quicker response of white blood cells in the fast-track group than in the conventional care group. We also found that blood levels of globulin, immunoglobulin G, and complement 4 on postoperative day 3 were higher in the fast-track group than in the conventional care group.

**Conclusions:**

Fast-track surgery accelerates clinical recovery and improves postoperative immunity after elective open surgery for colorectal carcinoma.

## Introduction

Fast-track surgery (FTS) is a promising comprehensive program for surgical patients. It aims to decrease the perioperative stress response to the surgical trauma, thereby leading to a decrease in complication rates after elective surgery [1, 2]. Numerous clinical trials have provided positive evidence of the benefits of utilizing FTS, including improving postoperative recovery, shortening the hospital stay, accelerating the return of gastrointestinal function, and reducing morbidity and mortality rates [3–6]. Some researchers believe that FTS also has positive effects on the human immune system, which may result in quicker recovery of postoperative immune function [7]. Nevertheless, few clinical studies or randomized clinical trial (RCT) results have reported the impact of FTS on human immunity. Therefore, based on the hypothesis and present evidence of the benefits of FTS, this prospective, randomized comparative study investigated the effects of FTS on postoperative clinical recovery and immunity in patients with colorectal carcinoma undergoing elective open surgery.

## Materials and methods

### Participants

This study was conducted in the Department of Gastrointestinal and Pancreatic Surgery, First Affiliated Hospital of Sun Yat-sen University from November 2008 to January 2009. The surgical procedures were performed by experienced surgeons (they had performed at least 200 colorectal procedures before participating in the study). Seventy patients who were clinically diagnosed as having colorectal carcinoma were assigned randomly to two groups comprising 35 patients each: FTS group and conventional care group. Inclusion criteria included: age ≥18 and ≤80 years, no preoperative chemotherapy or radiotherapy, American Society of Anesthesiologists (ASA) grade I/II, body mass index (BMI) 17.5–27.5 kg/m^2^, preoperative serum albumin ≥30 g/l. All of the patients underwent elective open colorectal resection with combined tracheal intubation and general anesthesia. Exclusion criteria included immune-related disease; primary diabetes mellitus or impaired glucose tolerance; hiatus hernia; gastroesophageal reflux disease (GERD); pregnancy; bowel obstruction; patients with difficult airway access (difficult to intubate); and drug intake, which might affect bowel movement and function. Patients also would be excluded if the following circumstances occurred: failure of thoracic epidural catheter insertion; intraoperative blood transfusion; patients who required a stoma; unresectable carcinoma.

The study protocol was approved by the Research Ethics Committee of the First Affiliated Hospital of Sun Yat-sen University (Guangzhou, China). Written informed consents were obtained from the patients and their families. This study was registered under chictr.org, identifier number ChiCTR-TRC-00000157.

### Interventions

The intervention protocols of the FTS group were as follows: normal meal until 10 p.m. the day before surgery; drink 250 ml of 5 % carbohydrate 2 h before surgery [8]; no routine nasogastric tube drainage; early as possible removal of urine and venous catheters (urinary catheter: removed when the patient became conscious and could be mobilized out of bed; deep venous catheter: removed when vital signs were stable); oral feeding started 6–12 h after surgery, following a stepwise plan from oral liquid nutrition to normal diet. Ensure (400 g; Abbott, Chicago, IL, USA) was applied as oral nutrition and was mixed with water for 1 Kcal/ml. The oral feeding plan was as follows: 6–12 h after surgery, Ensure mixture, 30–50 ml every 1–2 h; postoperative day (POD) 2 and afterward, Ensure mixture, 100–200 ml every 2–3 h, plus semi-fluids according to the patient’s tolerance. Mobilization was encouraged from the night of the operation. Patients were encouraged to meet predefined mobility targets over the postoperative days.

The intervention protocols of the conventional group were as follows: normal meal until 10 p.m. the day before surgery, routine use of nasogastric tube drainage, and oral intake initiated on return to normal gastrointestinal function (bowel sounds or flatus) following a stepwise plan from oral liquid nutrition (Ensure 400 g) to a normal diet. Patients were sat up and assisted to mobilize on POD 1, but they were not aggressively mobilized until discontinuation of the thoracic epidural. Urinary catheters were removed following epidural catheter removal.

The same interventions were applied in both groups: Routine bowel preparation was done with gentamicin and metronidazole. Polyethylene glycol electrolyte powder (HYGECON, Jiangxi, China) was used as a laxative. Other measures included prophylactic use of antibiotics; avoidance of long-acting opioids; intraoperative maintenance of normothermia with an upper-body forced-air heating cover; a midline incision of minimal length; intraoperative and postoperative fluid restriction; no routine use of abdominal drains; the combination of continuous epidural mid-thoracic local anesthetics plus nonsteroidal antiinflammatory drugs (NSAIDs) to control postoperative pain. Postoperative blood glucose was controlled with the fasting blood glucose (FBG) level maintained at <12 mmol/l. Administration of any blood product was unacceptable, as was giving any agent that could affect immunity. Total postoperative calorie administration was controlled in the range of 25–30 Kcal/kg per 24 h in both groups.

Discharge criteria included the following: normal body temperature; independently mobile; return to normal gastrointestinal function (defecation at least once); normal oral diet, no need for parenteral nutrition; controllable pain with oral analgesia; willing to go home. Patients were readmitted at the request of the primary care physician or if the patient made direct contact with the hospital describing deteriorating health at home. Patients were followed up within 1 month after discharge (follow-up by telephone every 3 days during the first 2 weeks, once a week during the last 2 weeks). The patient was told that the researcher should be informed promptly if the patient had any discomfort.

Both groups were protocol-driven, with checklists for patients, nursing staff, and surgical staff to help maintain compliance. Teaching sessions and dummy runs were held before trial commencement to clarify potential points of confusion and reduce protocol violations. Patients were admitted to one of two nursing areas depending on the results of randomization. Although the interventions were protocol-driven, a geographically separate location was considered desirable to minimize protocol contamination.

### Measurements

Patients’ preoperative self-feelings were evaluated before anesthesia induction (e.g., thirsty, hungry). Anesthesia-related complications were measured. Intraoperative measurements were carefully recorded in detail, including surgical procedures, blood loss, fluid transfusion, and blood transfusion, among others. The return of normal gastrointestinal function (time to first bowel sounds/flatus, defecation, initiation of soft diet), hospital stay, and complications were recorded postoperatively. Blood tests [white blood cell (WBC) count, liver function tests (LFTs), serum biochemistry, humoral immunologic index] were performed on appointed days. The humoral immunologic factors tested in our study included serum globulin, immunoglobulin G (IgG), immunoglobulin M (IgM), immunoglobulin A (IgA), complement 3 (C3), and complement 4 (C4).

Experimental blood tests were performed on the morning of the operation and on PODs 1, 3, and 7. All blood samples were taken from peripheral veins at 6 a.m., before breakfast. We also took blood samples to test the WBC count at the end of surgery.

### Sample size, randomization, and implementation

The intention of our study was to detect possible changes of human immunity on the basis of clinical benefits. Like many other clinical studies, we selected the length of hospital stay (LOS) as the main endpoint. On the basis of previous data for postoperative LOS, (10.38 days on average) for patients undergoing major colonic surgery at our institution, we calculated that 35 patients in each group would be required to detect a 30 % reduction in postoperative LOS with an α level of 0.05 and a β level of 0.01.

Patients were informed about the aims and details of this study. Patients signed consent forms after the study was explained. Block randomization was computer-generated. Eligible patients were randomly assigned in a 1:1 ratio. The investigators who designed the study prepared the envelopes and assigned participants to their groups but had no contact with the patients throughout the study. The investigator recruiting the patients, administering the interventions, and evaluating the outcomes had no role in the randomization process.

### Statistical analyses

Data were analyzed using SPSS for Windows 13.0 (SPSS, Chicago, IL, USA). Numerical variables were expressed as the mean ± SD unless otherwise stated. Categoric variables were expressed by a constituent ratio or rate. Differences between the two groups were tested using a two-tailed Student’s *t* test for normally distributed data and the Wilcoxon test for noncontinuous variables. The χ^2^ test and Fisher’s exact test were used to compare discrete variables. A value of *p* < 0.05 was considered statistically significant.

Compared with our primary protocol, we made a modification to the enrollment of participants before trial commencement, which initially intended to enroll patients with gastrointestinal tumors other than colorectal cancer. The aim was to control the homogeneity of the patients and thus control bias. The sample size decreased from 60 to 35 accordingly. The Research Ethics Committee of the First Affiliated Hospital of Sun Yat-sen University (Guangzhou, China) approved all the changes.

## Results

**Fig. 1:**
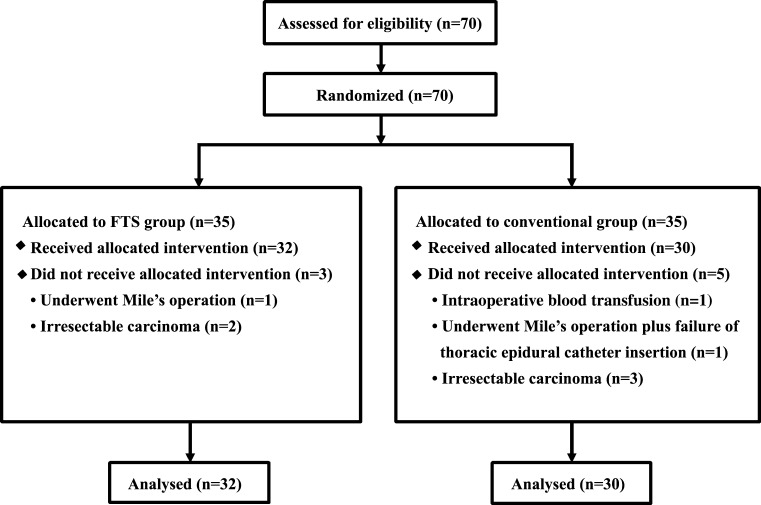
Patient flow throughout the study. *FTS* fast-track surgery

In all, 62 patients finished the study, including 32 patients in the FTS group and 30 in the conventional care group. Three patients were excluded from the FTS group and five patients from the conventional care group. (Fig. 1) Patients in the two groups had comparable preoperative baseline characteristics, including sex, age, serum hemoglobin and albumin levels, and body mass index (Table 1).

**Table 1:** Patients’ preoperative/intraoperative characteristics

Characteristic	FTS	Conventional
	(*n* = 32)	(*n* = 30)
Age (years)	57.2 ± 11.70	59.5 ± 12.10
Sex (no.)		
Male	20 (62.5 %)	22 (73.3 %)
Female	12 (37.5 %)	8 (26.7 %)
Body mass index (kg/m^2^)	22.25 ± 2.45	21.69 ± 2.48
Hemoglobin (g/l)	125.8 ± 18.8	129.8 ± 20.1
Albumin (g/l)	40.84 ± 2.95	40.67 ± 3.58
Preoperative feeling (no.)		
Thirsty*	2 (6.3 %)	23 (76.7 %)
Hungry*	5 (15.6 %)	20 (66.7 %)
Operating time (min)	209 ± 40.1	196 ± 50.6
Blood loss (ml)	150 ± 100^a^	200 ± 100^a^
Fluid transfusion (ml)	2800 ± 500^a^	2925 ± 500^a^
Type of surgery (no.)		
Right hemicolectomy	6 (18.7 %)	7 (23.3 %)
Left hemicolectomy	2 (6.3 %)	3 (10.0 %)
Sigmoidectomy	6 (18.7 %)	7 (23.3 %)
Dixon operation	18 (56.3 %)	13 (43.3 %)
Tumor staging (no.)		
TNM classification		
I	5 (15.6 %)	7 (23.3 %)
II	18 (56.3 %)	16 (53.3 %)
III	9 (28.1 %)	7 (23.3 %)
Dukes classification		
A	5 (15.6 %)	7 (23.3 %)
B	18 (56.3 %)	16 (53.3 %)
C1	3 (9.4 %)	2 (6.7 %)
C2	6 (18.7 %)	5 (16.7 %)

*FTS* fast-track surgery

*Variables* were expressed as the median ± quartile

^a^Not subject to normal distribution

* *p* < 0.05

No statistically significant differences were detected between the two groups regarding the operating time, blood loss or fluid transfusion during the operation, surgical procedure, or tumor staging. However, patients in the FTS group did experience significantly less discomfort in terms of hunger and thirst (Table 1).

### Postoperative clinical parameters

Patients in the FTS group showed significantly accelerated recovery of gastrointestinal function compared to that of the conventional care group in terms of time to first bowel sounds/flatus (2 ± 1 vs. 4 ± 2 days, *p* < 0.05), defecation (3.84 ± 1.63 vs. 6.44 ± 2.53 days, *p* < 0.05), and initiation of soft diet (4.0 ± 2.0 vs. 8.2 ± 2.16 days, *p* < 0.05). Postoperative hospital stay was significantly shorter in the FTS group than in the conventional care group (6.0 ± 1.0 vs. 11.7 ± 3.82 days, *p* < 0.05).

Although no statistically significant differences were found for the surgical site infection (SSI) rate (2/30 vs. 1/32, *p* = 0.6066), pneumonia (1/30 vs. 0/32, *p* = 0.4839), and intestinal dysbiosis (5/30 vs. 1/32, *p* = 0.0986) between conventional care and FTS groups, patients in the FTS group had a significantly lower rate of total infectious complications than did the conventional group (8/30 vs. 2/32, *p* < 0.05). No statistically significant differences were found for noninfectious complications between the conventional care and FTS groups (4/30 vs. 4/32, *p* = 1.0000), including vomiting (1/30 vs. 3/32, *p* = 0.6132), stress ulcer (1/30 vs. 0/32, *p* = 0.4839), arrhythmia (1/30 vs. 0/32, *p* = 0.4839), and urine distension (1/30 vs. 1/32, *p* = 1.0000). No anastomotic leakage, anastomotic bleeding, abdominal infection, anesthesia-related complications, or hospital readmissions due to complications were detected in either group.

### White blood cell count

A statistically significant difference was found regarding the WBC count at the end of surgery, with a higher WBC count found in the FTS group than in the conventional care group (Table 2). No statistically significant difference was detected in levels of WBC count on the morning of the day of the operation. The tendencies for WBC count change were different in the two groups. In the FTS group, the WBC count rose quickly to the highest point at the end of surgery and then dropped gradually to a normal level on POD 7. In conventional group, the WBC count quickly rose to a high level at the end of surgery and fluctuated at that high level until POD 7 (Table 2). Thus, the FTS group had a quicker WBC response, including rising and dropping counts, than the conventional care group.

**Table 2:** White blood cell count

Time	FTS	Conventional	Statistic	*p*
	(*n* = 32)	(*n* = 30)	(Z)	
Before surgery	6.94 ± 2.08	6.21 ± 2.35^a^	−1.5264	0.1269
End of surgery	12.38 ± 3.16	10.65 ± 2.58^a^	−2.1785	0.0294
POD 1	11.84 ± 2.57	10.15 ± 2.57^a^	−1.3838	0.1664
POD 3	10.45 ± 5.28^a^	11.08 ± 2.23	0.2831	0.7771
POD 7	8.55 ± 5.70^a^	10.25 ± 5.52^a^	1.3172	0.1878

*POD* postoperative day

*Variables* were expressed as the median ± quartile

^a^Not subject to normal distribution

### Humoral immunologic parameters

No statistically significant differences were detected in preoperative levels of serum globulin, IgG, IgM, IgA, C3, or C4 between the two groups. On POD 3, statistically significant differences were found in levels of serum globulin, IgG, and C4, with the FTS group having higher levels than in the conventional group (Table 3).

**Table 3:** Serum level of humoral immunologic factors

Factor and time	FTS	Conventional	Statistic	*p*
	(*n* = 32)	(*n* = 30)		
Globulin (g/l)				
Before surgery	27.6 ± 4.0	26.4 ± 4.9	T = 0.9174	0.3643
POD 1	21.6 ± 3.4	19.6 ± 3.5	T = 1.8923	0.0657
POD 3	24.1 ± 2.4	22.1 ± 3.3	T = 2.3257	0.0252
POD 7	27.5 ± 3.7	26.6 ± 4.5	T = 0.721	0.4753
IgG (g/l)				
Before surgery	13.76 ± 3.35	11.81 ± 2.66	T = 1.98	0.0559
POD 1	10.35 ± 2.61	8.99 ± 2.32	T = 1.6496	0.1080
POD 3	10.79 ± 2.39	8.66 ± 2.09	T = 2.8828	0.0067
POD 7	13.27 ± 2.82	11.29 ± 3.09	T = 2.0102	0.0524
IgA (g/l)				
Before surgery	2.51 ± 1.08^a^	2.44 ± 1.07^a^	Z = –0.3381	0.7353
POD 1	2.03 ± 0.65	1.88 ± 0.59	T = 0.7213	0.4754
POD 3	2.33 ± 0.66	2.07 ± 1.00^a^	Z = −1.0525	0.2926
POD 7	2.98 ± 0.96	2.94 ± 1.07	T = 0.1393	0.8900
IgM (g/l)				
Before surgery	1.01 ± 0.38	1.04 ± 0.41	T = −0.2270	0.8217
POD 1	0.71 ± 0.29	0.78 ± 0.27	T = −0.6878	0.496
POD 3	0.78 ± 0.36	0.75 ± 0.31	T = 0.2685	0.7898
POD 7	1.09 ± 0.59^a^	1.52 ± 0.85	Z = 0.9727	0.3307
C3 (g/l)				
Before surgery	1.08 ± 0.17	0.99 ± 0.20	T = 1.5005	0.142
POD 1	0.82 ± 0.14	0.76 ± 0.17	T = 1.2622	0.215
POD 3	0.82 ± 0.15	0.73 ± 0.20	T = 1.5935	0.1198
POD 7	0.99 ± 0.23	0.88 ± 0.26	T = 1.4533	0.1551
C4 (g/l)				
Before surgery	0.29 ± 0.10	0.26 ± 0.07	T = 1.23	0.2274
POD 1	0.21 ± 0.08	0.19 ± 0.06^a^	Z = −1.0749	0.2824
POD 3	0.24 ± 0.09	0.17 ± 0.05^a^	Z = −2.1099	0.0349
POD 7	0.28 ± 0.12	0.22 ± 0.10^a^	Z = −1.7498	0.0801

*Ig* immunoglobulin, *C3*, *C4* complement 3 and 4, respectively

*Variables* were expressed as the median ± quartile

^a^Not subject to normal distribution

No statistically significant differences were detected in the postoperative levels of serum IgM, IgA, or C3 between the two groups (Table 3). There were also no statistically significant differences in the recovery rates of all factors (Table 4).

**Table 4:** Recovery rate of humoral immunologic factors

Factor and time	FTS	Conventional	Statistic	*p*
	(*n* = 32)	(*n* = 30)		
Globulin (%)				
POD 1	78.9 ± 10.9	73.3 ± 9.1	T = 1.7335	0.0907
POD 3	89.0 ± 15.0	85.0 ± 12.3	T = 0.9349	0.3555
POD 7	98.8 ± 12.6	102.1 ± 15.4	T = –0.7579	0.4532
IgG (%)				
POD 1	75.5 ± 9.2	74.2 ± 11.0	T = 0.3964	0.6942
POD 3	78.9 ± 11.0	74.0 ± 10.2	T = 1.3963	0.1714
POD 7	97.3 ± 13.1	94.9 ± 10.7	T = 0.6061	0.5484
IgA (%)				
POD 1	77.0 ± 9.6	75.4 ± 11.3^a^	Z = −0.2202	0.8257
POD 3	87.2 ± 10.1	83.9 ± 11.7	T = 0.9406	0.3532
POD 7	110.8 ± 13.7	113.3 ± 16.2	T = −0.5083	0.6145
IgM (%)				
POD 1	70.8 ± 9.1	71.7 ± 9.9	T = −0.2985	0.7671
POD 3	74.8 ± 12.1	73.6 ± 11.9	T = 0.3090	0.7591
POD 7	112.2 ± 58.1^a^	115.0 ± 57.5^a^	Z = 0.3191	0.7497
C3 (%)				
POD 1	76.7 ± 9.8	75.1 ± 9.6	T = 0.4969	0.6223
POD 3	76.7 ± 15.3	75.2 ± 18.0	T = 0.2763	0.7839
POD 7	94.1 ± 24.9	87.0 ± 20.9^a^	Z = −0.7445	0.4566
C4 (%)				
POD 1	73.0 ± 8.6	72.1 ± 10.4	T = 0.2935	0.7708
POD 3	82.5 ± 14.6	77.5 ± 20.3	T = 0.8711	0.3895
POD 7	98.5 ± 24.6	87.2 ± 30.6^a^	Z = −1.3221	0.1861

*Variables* were expressed as median ± quartile

^a^Not subject to normal distribution

## Discussion

Numerous clinical trials have provided positive evidence of the benefits of utilizing FTS [3–6]. However, most of the FTS studies or reviews/meta-analysis intended only to determine the clinical impact of FTS [3–6, 9–12], with only a few studies evaluating the impact of FTS on human immunity. The aim of the present RCT was to evaluate prospectively the clinical and immunologic results of fast-track colorectal surgery.

### Interpretation

In this study, the principal differences between the two arms concern a shorter period of preoperative starvation, early removal of catheters, early oral feeding, and earlier mobilization. Our findings indicate that FTS leads to a significantly faster recovery of gastrointestinal function, as indicated by time to first flatus, bowel movements, and initiation of a soft diet. In addition, patients in the FTS group suffered significantly fewer infectious complications without increasing noninfectious ones. In agreement with these clinical advantages, we observed a significant decrease in the postoperative length of hospital stay in fast-track patients.

As far as the immunologic effects are concerned, one study demonstrated that, compared to carbohydrate intake before surgery, fasting may abate the expression of monocyte HLA-DR postoperatively [13]. Another study showed that the use of FTS perioperatively enhanced the human body’s cellular immunologic ability [i.e., T cells, Th cells, natural killer (NK) cells] [7]. In this clinical trial, we found that the FTS group had a higher WBC count than did the conventional care group at the end of surgery. In addition, the FTS group had a quicker WBC response, including rising and dropping counts, than the conventional care group. Although we did not test the differences in the WBC subgroups, the changes that were seen represented some degree of cell-mediated immunity difference between the two groups.

Until now, no studies have reported the effect of FTS on human humoral immunity. Our results indicated that FTS accelerated the recovery of serum globulin. As we know, immunoglobulin and complement are two vital elements of globulin [14, 15]. Our findings showed that FTS group had significantly higher levels of serum IgG and C4 on POD 3. With its high affinity and wide distribution, IgG is the most abundant immunoglobulin in the blood and extracellular fluid, playing a major role in the immune response to fight infectious pathogens [14]. The complement system is an important component of the innate immune system. The major functions of the complement system include direct killing of microorganisms; opsonization of microorganisms for phagocytosis, chemotaxis, and activation of leukocytes and mast cells; and processing of immune complexes and regulation of antibody production by B cells. C4 plays a key role in the classic and lectin pathways, which are the two major pathways to activate the complement system. Complement also plays an important role in adaptive immunity involving T and B cells, which help in the elimination of pathogens [14–16].

The correlation of clinical findings with the immune parameters indicates that the beneficial clinical data reported here are associated with better-preserved immunity. However, the mechanisms need to be further studied.

### Generalizability and limitations of the study

We found the fact that FTS has a positive impact on WBCs, but it is unfortunate that we did not test the changes in the WBC subgroups. Our results did not detect statistically significant changes in serum IgA, IgM, or C3 between the two groups. However, this does not mean that FTS has no effects on these factors, which may be explained by the time that blood samples were taken or the relatively small number of cases in our trial. Also, we did not intend to identify the precise mechanism by which FTS affects the immunity.

Patients recruited in our study were relatively healthy and underwent elective open colorectal resection. The conclusions of this study may not be able to be extrapolated to patients who do not meet the same inclusion criteria.

## Conclusions

Our study suggests that fast-track surgery accelerates clinical recovery and improves postoperative immunity in patients undergoing elective open surgery for colorectal carcinoma. The precise mechanism of how FTS affects the immunity needs further study.

## References

[1] Wilmore DW, Kehlet H (2001). Management of patients in fast track surgery. Br Med J.

[2] Kehlet H (2008). Fast-track colorectal surgery. Lancet.

[3] Khoo CK, Vkkery CJ, Forsyth N (2007). A prospective randomized controlled trial of multimodal perioperative management protocol in patients undergoing elective colorectal resection for cancer. Ann Surg.

[4] Ionescu D, Iancu C, Ion D (2009). Implementing fast-track protocol for colorectal surgery: a prospective randomized clinical trial. World J Surg.

[5] Varadhan KK, Neal KR, Dejong CH (2010). The enhanced recovery after surgery (ERAS) pathway for patients undergoing major elective open colorectal surgery: a meta-analysis of randomized controlled trials. Clin Nutr.

[6] Berberat PO, Ingold H, Gulbinas A (2007). Fast track: different implications in pancreatic surgery. J Gastrointest Surg.

[7] Wichmann MW, Eben R, Angele MK (2007). Fast track rehabilitation in elective colorectal surgery patients: a prospective clinical and immunological single-center study. ANZ J Surg.

[8] Yang DJ, He YL, Cai SR (2009). Effects of drinking fluid before anesthesia on gastric fluid volume and pH in patients with colorectal cancer. JDO (Electronic version).

[9] Kehlet H, Wilmore DW (2008). Evidence-based surgical care and the evolution of fast-track surgery. Ann Surg.

[10] Mastracci TM, Cohen Z, Senagore A (2008). Canadian Association of General Surgeons and American College of Surgeons Evidence-Based Reviews in Surgery. 24. Fast-track programs in colonic surgery: systematic review of enhanced recovery programmes in colonic surgery. Can J Surg.

[11] Lassen K, Soop M, Nygren J (2009). Consensus review of optimal perioperative care in colorectal surgery: enhanced recovery after surgery (ERAS) group recommendations. Arch Surg.

[12] Olsén MF, Wennberg E (2011). Fast-track concepts in major open upper abdominal and thoracoabdominal surgery: a review. World J Surg.

[13] Melis GC, van Leeuwen PA, von Blomberg-van der BM (2006). A carbohydrate-rich beverage prior to surgery prevents surgery-induced immunodepression: a randomized, controlled, clinical trial. JPEN J Parenter Enteral Nutr.

[14] Delves PJ, Roitt IM (2000). The immune system. First of two parts. N Engl J Med.

[15] Dunkelberger JR, Song WC (2010). Complement and its role in innate and adaptive immune responses. Cell Res.

[16] Reuschenbach M, von Knebel Doeberitz M, Wentzensen N (2009). A systematic review of humoral immune responses against tumor antigens. Cancer Immunol Immunother.

